# Infrared Spectroscopic Verification of a α-Helical Collagen Structure in Glutaraldehyde-Free Crosslinked Bovine Pericardium for Cardiac Implants

**DOI:** 10.3390/life12122035

**Published:** 2022-12-06

**Authors:** Cindy Welzel, Ulla König, Anett Jannasch, Klaus Matschke, Sems-Malte Tugtekin, Claudia Dittfeld, Gerald Steiner

**Affiliations:** 1 Department of Cardiac Surgery, Carl Gustav Carus Faculty of Medicine, Technische Universität Dresden, Heart Centre Dresden, 01307 Dresden, Germany; 2 Department of Medical and Biotechnological Applications, Fraunhofer Institute for Organic Electronics, Electron Beam and Plasma Technology, 01277 Dresden, Germany; 3 Department of Anaesthesiology and Critical Care Medicine, Clinical Sensoring and Monitoring, Faculty of Medicine, Technische Universität Dresden, 01307 Dresden, Germany

**Keywords:** heart valve bioprostheses, calcific aortic valve disease, biomineralization, GA-fixation, SULEEI-preparation, IR spectroscopic imaging

## Abstract

The degeneration of heart valve bioprostheses due to calcification processes is caused by the intercalation of calciumhydroxyapatite in pericardium collagen bundles. Variations of the protein secondary structure of biomaterials according to preparation are relevant for this mineralization process and thus the structural characterization of innovative bioprostheses materials is of great importance. The gold standard for prostheses preparation is glutaraldehyde (GA)-fixation of bovine pericardium that adversely promotes calcification. The novel GA-free SULEEI-treatment of bovine pericardium includes decellularization, UV-crosslinking, and electron beam sterilization. The aim of this study is the structural characterization of SULEEI-treated and GA-fixed bovine pericardium. IR spectroscopic imaging combined with multivariate data and curve fit analysis was applied to investigate the amide I and amide II regions of SULEEI-treated and GA-fixed samples. The spectroscopic images of GA-fixed pericardial tissue exhibited a generally high content of amine groups and side chains providing nucleation points for calcification processes. In contrast, in SULEEI-treated tissue, the typical α-helical structure was retained and was supposed to be less prone to deterioration.

## 1. Introduction

Valvular heart disease (VHD) is a major contributor to loss of physical function, quality of life and longevity [1]. Calcific aortic stenosis is the most common manifestation of VHD. It affects approximately 9 million people worldwide and deaths from calcific aortic stenosis have continued to rise in the past 20 years [1]. To date the only option to treat affected patients is the surgical replacement of the aortic valve by an artificial mechanical or biological prosthetic valve. Mechanical prostheses have the disadvantage of thrombus formation and patients need life-long anticoagulation [2,3]. Therefore, biological prostheses made out of bovine pericardium or porcine aortic valves are used more frequently [4]. To date, the gold standard of bioprostheses preparation is the fixation of bovine pericardium with glutaraldehyde (GA). This crosslinks collagen fibers, reduces immunogenicity, and makes the material sterile simultaneously [5,6]. The remaining aldehyde groups and variations in the collagen protein structure promote calcification processes, which lead to early loss-of-function and the need for re-operation after 10 to 15 years [6,7,8,9,10,11].

One main research focus is thus the development of GA-free preparation strategies to improve the lifespan of bioprostheses [6]. The procedure of the stabilization and sterilization of acellular pericardial scaffolds combining photo-initiated ultraviolet cross-linking with low-energy electron irradiation (SULEEI) is an innovative alternative preparation strategy of the bovine pericardium [12,13]. In the three-step SULEEI protocol, bovine pericardium is firstly decellularized to remove DNA and cellular material, generating a cell-free bovine pericardial extracellular matrix (ECM). This is a critical step as it prevents immune reactions caused by xenogenic cellular material such as the epitopes galactose-alpha-1,3-galactose (α-Gal) and n-glycolneuraminic acid (Neu5gc) [5,14]. Efficient decellularization is evaluated by the absence of nuclei in H&E staining, as well as a DNA threshold below 50 ng double strand DNA per mg ECM dry weight and with a fragment length of <200 bp [15]. 

At the same time, it is favored that ECM components and structure are preserved during this procedure. A variety of chemical and biological agents as well as physical methods for decellularization have been established [15,16]. However, all these methods lead to a disruption in ECM structure and composition [15,17,18,19]. It was shown that decellularization is attended by a reduction in glycosaminoglycans (GAGs) and elastin, two of the major ECM components providing structural and biochemical support, as well as enabling proper mechanical function [20,21]. Therefore, developing gentle decellularization methods that retain GAGs and elastin as well as collagen is still one subject of research with great importance. In the second step of the SULEEI protocol, the cell-free pericardial material is crosslinked using photo-initiated ultraviolet (UV) irradiation to improve the mechanical stability. This technology was developed by Wollensak et al., 2003, and is used for corneal crosslinking for the treatment of keratoconus [22]. It is based on the photosensitizer riboflavin, which is excited by a UV light at 370 nm, creating free radicals that crosslink collagen fibers [23]. In the last SULEEI step, the material is sterilized and even further crosslinked by low-energy electron irradiation [12,13]. Irradiation with low-energy electrons is, in comparison with high-energy electrons or gamma irradiation, gentler to the protein structure [12,24], but it still provides appropriate sterilization according to legal regulations (c.f. German DIN EN ISO 11137).

The primary focus in prostheses material research is biomechanical testing accompanied by biochemical evaluation of the ECM composition [5]. The biomechanical behavior of SULEEI-treated pericardium has already been investigated and is comparable to conventionally used GA-fixed pericardium [13,25]. The biochemical evaluation of ECM components is often destructive and results in changes, for example, in experimental processing during histology. Therefore, molecular spectroscopy, in particular Fourier-Transform Infrared (IR) spectroscopy, was performed to study bovine pericardium because it is a convenient technique providing detailed structural information on a molecular level.

Until now, IR spectroscopy has been used to investigate mineralization and calcification processes of human aortic valve (AV) tissue [26,27,28]. For example, Mikroulis et al. compared calcific deposits from natural and bioprosthetic heart valves by IR spectroscopy and found that crystal growth proceeds in both types by the hydrolysis of calcium phosphate phases to hydroxyapatite (HAP) [26]. Prieto et al. also investigated calcific deposits from natural human heart valves and analyzed their chemical composition by IR spectroscopy. They discovered deposits with a high calcium/phosphate ratio, which were rich in magnesium and calcium phosphate, but also deposits with a low calcium/phosphate ratio, with low magnesium and high HAP contents [27]. Not only the calcification of aortic valves but also their structure can be investigated by IR spectroscopy. Jastrzebska et al. analyzed the different components, i.e., collagen and elastin, of the three layers of human heart valves and showed that IR spectroscopy is a suitable method for the structural characterization of heart valves on a molecular level [29]. In addition, it was shown that IR spectroscopy allows for the rapid non-invasive assessment of bioscaffolds to evaluate oxidation, degradation, and denaturation prior to transplantation [28].

An increased content of the β-sheet collagen secondary structure has been proposed to be correlated with calcification of ECM in human aortic valve tissue, but also in mineralized bioprosthesis [30]. In addition, this was observed in calcified and non-calcified regions of bioprosthetic tissue based on bovine pericardium after patient re-operation, whereas protein signals in the mineralized area were only detectable in the border transition zones [30]. When investigating non-implanted bioprostheses, a more pronounced β-sheet characteristic was also found, leading to the conclusion that a pretreatment strategy such as incubation in GA may be responsible for a potential calcification event [30]. Therefore, GA-fixed bovine pericardium was investigated by IR-spectroscopy in the presented study quantifying the β-sheet vs. α-helix band characteristics in more detail. Additionally, SULEEI-treated pericardia were analyzed by IR-spectroscopy, as GA-free preparation protocols are the focus of intense investigation by various groups [5,6,31].

In this study, variations of the secondary structure of GA-fixed and SULEEI-treated pericardia were characterized and quantified by IR spectroscopic imaging in combination with multivariate data and curve fit analysis.

## 2. Materials and Methods

### 2.1. Tissue Preparation and Treatment

Bovine pericardia from two to eight years old cattle (*n* = 5) were kindly provided by a local slaughterhouse (Vorwerk Podemus, Dresden, Germany). Pericardia were dissected and washed in PBS to remove the connective tissue. The SULEEI treatment [12,13] was performed at the Fraunhofer Institute for Organic Electronics, Electron Beam, and Plasma Technology FEP (Dresden, Germany). Briefly, the bovine pericardia were at first decellularized according to Roosens et al. [32], secondly they were incubated in a 2% dextran solution (Carl Roth GmbH & Co. KG, Karlsruhe, Germany) containing 0.1% riboflavin (Serva Electrophoresis GmbH, Heidelberg, Germany) prior to UV irradiation to crosslink collagen fibers, and lastly the pericardia were sterilized by low-energy electron irradiation (Table 1). As a control material, conventionally GA-fixed bovine pericardia (in 0.625% GA in 4.863 g/l HEPES supplemented with 2.65 g/L MgCl_2_*6 H_2_O, 4.71 g/L NaCl, pH 7.4 for 3 h at room temperature) were prepared. Pericardial thickness was measured using a thickness gauge Model FD50 (Käfer Messuhrenfabrik GmbH and Co. KG, Villingen-Schwenningen, Germany).

Verification of the sample sterility performed as described before (incubation in a CASO bouillon (Carl Roth GmbH & Co. KG, Karlsruhe, Germany) at 30 °C for 14 days) was the basis for the sample implementation in this study [12].

### 2.2. FT-IR Spectroscopy

GA-fixed and SULEEI-treated pericardia were embedded in cryosectioning media Tissue-Tek^®^ O.C.T.™ Compound (Sakura Finetek GmbH, Umkirch, Deutschland) and frozen at −20 °C. Cryosections with a thickness of 5 µm were transferred onto a CaF_2_ window. IR spectroscopic images across the whole pericardium cross-section were collected in transmission mode using an FT-IR spectrometer (Vertex 70) coupled with an infrared microscope (Hyperion 3000; both from Bruker Optik GmbH, Ettlingen, Germany) and a nitrogen-cooled HgCdTe 64 × 64 focal plane array detector (FPA) (Santa Barbara Focal Plane, Santa Barbara, CA, USA). The FPA detector was equipped with a Ge window which absorbed IR light below 950 cm^−1^. The 15-fold Cassegrainian objective imaged a sample area of approx. 250 × 250 µm. Compositions of individual IR spectroscopic images of different samples were captured subject to the size of the area investigated. Reference spectroscopic images were recorded from the pure CaF_2_ window before the tissue sections were investigated. For all of the measurements, a total of 150 interferograms (scans) were co-added. The interferograms were Fourier transformed, applying a Happ-Genzel apodization and a zero filling factor of 1. Spectra at a resolution of 6 cm^−1^ of the sample image were rationed against the spectra of the reference image taken from the pure CaF_2_ window and were transferred to absorbance values. This spectral resolution was chosen to improve the signal-to-noise ratio in order to reduce the size of the spectral data set and to ensure that all prominent bands, even those with medium intensity, appeared clearly in the spectrum.

### 2.3. Data Processing and Multivariate Analysis

The spectral data were analyzed using the MATLAB Package (Version 9, Math Works Inc., Natick, MA, USA). The so-called fingerprint region between 950 cm^−1^ and 1800 cm^−1^ was considered. Data preprocessing involved the removal of outliers, i.e., spectra that are obviously not associated with soft tissue or spectra with a maximum absorbance value of the amide I band larger than 0.015. For further analysis, only the amide I and II region between 1480 cm^−1^ and 1705 cm^−1^ was selected and spectra within this region were offset corrected. Cluster analysis was then performed using the k-means function of the Statistics Toolbox of MATLAB. For cluster analysis, the spectroscopic data sets of all SULEEI and GA samples were merged to multidata files (SULEEI and GA). The spectra were area normalized to compensate variations of the sample thickness and density of the tissue. According to the elbow criterion [33], the number of clusters was set to 15. The centroid spectra of the 15 selected clusters were subsequently subjected to a curve fit analysis to determine the proportions of secondary structure units.

The curve fit analysis was performed with the program Grams (version 8.0, ThermoF Electron Cooperation, Waltham, MA, USA), where 14 subbands with a Gaussian profile were given. The number of 14 subbands was determined using the fit routine and the function implemented in it to estimate the number of subbands. For the subsequent quantitative analysis, the spectra were off-set corrected and area normalized in the analyzed spectral range. No additional baseline correction was performed. The position of the subbands was specified with a tolerance range of ±3 cm^−1^. Further constraints on the fit parameters concerned the minimum band height of h > 0 and the range of the band half-width from 10 cm^−1^ to 25 cm^−1^. The three subbands with the most prominent differences between GA-fixed and SULEEI-treated samples were visualized in RGB plots. In order to achieve the highest possible contrast and at the same time to enable a comparative evaluation, the subbands were min/max normalized. This means that all 2 × 14 subbands with the same spectral position were normalized in such a way that the largest band area was set to one and that of the smallest was set to zero.

### 2.4. Histological Staining

Pericardia were fixed in 4% formalin (SAV Liquid Production GmbH, Flintsbach am Inn, Germany) in PBS, embedded in paraffin, and sectioned with a thickness of 3 µm. Histological evaluation was performed to analyze the morphological differences between GA-fixed and SULEEI-treated pericardia. Nuclei and collagen were visualized using H&E staining according to the standard protocol. Images were acquired using a slide scanner (Axio ScanZ.1 by Zeiss, Jena, Germany).

### 2.5. Statistical Analysis

Statistical analysis was performed using GraphPad Prism version 9 (GraphPad Software, La Jolla, CA, USA). Statistically significant differences (* *p* < 0.05, ** *p* < 0.01, *** *p* < 0.001) of normally distributed data sets were analyzed using *t*-test.

## 3. Results

**Figure 1:**
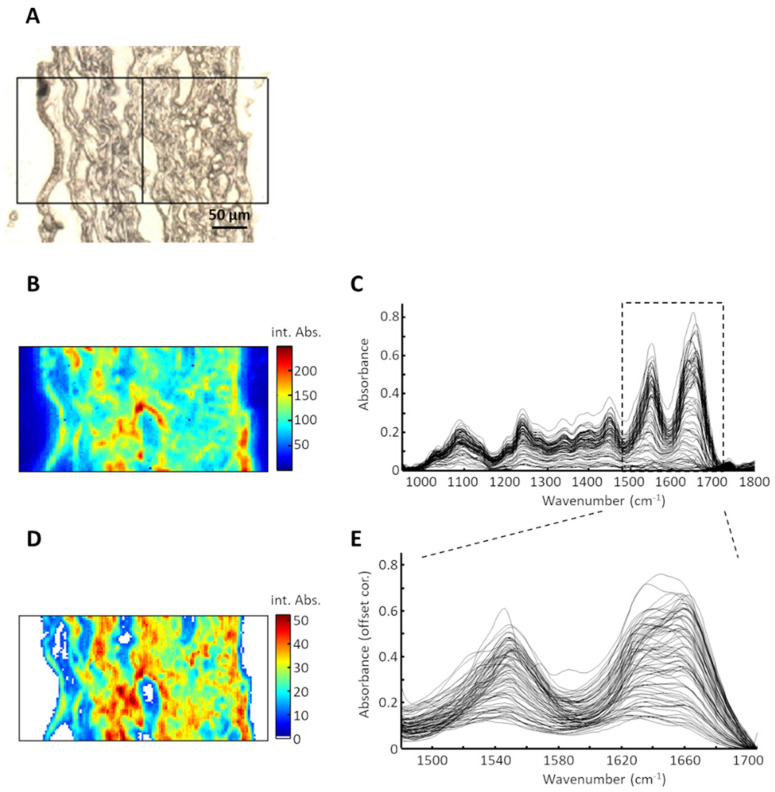
Processing of spectral data obtained by IR spectroscopy. (**A**) Brightfield image of bovine pericardium cross-section with the scan area. (**B**) Intensity map of the analyzed sample region. (**C**) Plot of raw spectra obtained from IR spectroscopy. (**D**) Intensity map of the spectral region 1480 cm^−1^–1705 cm^−1^ after the selection of outliers (represented by white pixels). (**E**) Plot of the selected and offset corrected spectra. IR—infrared.

IR spectroscopic imaging was used to investigate the effect of the SULEEI-treatment on the collagen secondary structure in comparison with GA-fixed tissue. Figure 1A shows the microscopic image of a sample section as an example. The 2 × 1 composite IR spectroscopic image is shown in Figure 1B. The contrast was calculated from the integral absorbance between 950 cm^−1^ and 1800 cm^−1^ (brightfield image). The plot of the recorded raw spectra in Figure 1C reveals the typical profile of the IR spectra from the tissue. Absorption bands of amide I (1600 cm^−1^–1700 cm^−1^) and amide II (1500 cm^−1^–1600 cm^−1^) dominated the spectral profile. The weaker absorption bands around 1400 cm^−1^–1460 cm^−1^ arose from CH_X_ groups of proteins and lipids, whereas the absorption band around 1230 cm^−1^ represented the amide III band. The broad absorption band between 1000 cm^−1^–1150 cm^−1^ mainly originated from carbohydrates, carbonate, and phosphate groups. Figure 1D shows the spectroscopic brightfield image in the spectral range of 1480 cm^−1^–1705 cm^−1^. White pixels indicate spectra that were detected as outliers and removed from the data set. The corresponding spectra are plotted in Figure 1E. The amide I band in particular provides information about the protein secondary structure.

**Figure 2:**
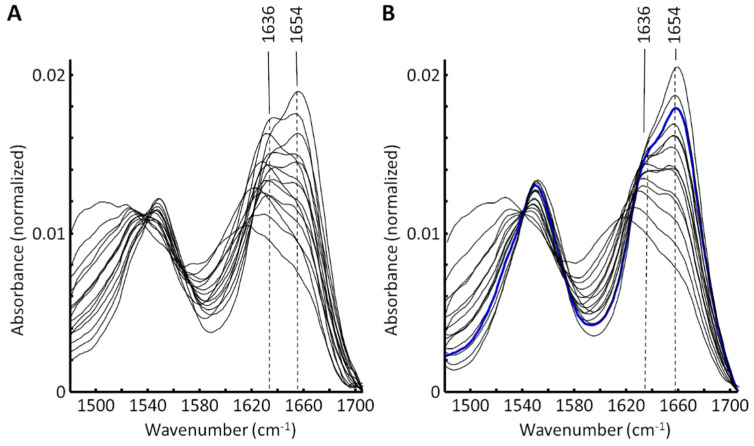
Centroid spectra after cluster analysis of GA-fixed (**A**) and SULEEI-treated bovine pericardium samples (**B**). The resulting subbands after curve fitting are exemplarily shown below (Figure 3) for the blue spectrum in (**B**). GA—Glutaraldehyde, SULEEI—Stabilization and sterilization of acellular pericardial scaffolds combining photo-initiated Ultraviolet cross-linking with Low-Energy Electron Irradiation.

In order to estimate the proportions of the secondary structure units, k-means cluster analysis was performed. This multivariate statistical method clustered spectra based on their similarity and the corresponding centroid spectra allowed for drawing conclusions about the dominant secondary structure in each cluster. For example, α-helical substructures were absorbed at ~1654 cm^−1^, whereas β-sheet substructures had their absorption maxima at ~1636 cm^−1^ [34]. Figure 2 shows the centroid spectra for GA-fixed (A) and SULEEI-treated tissue samples (B). Bands around 1636 cm^−1^ and 1654 cm^−1^ were present in both tissues. While in GA-fixed samples the band at 1636 cm^−1^ is clearly prominent in several centroid spectra, the signal appeared much weaker in the centroid spectra of the SULEEI-treated samples.

**Figure 3:**
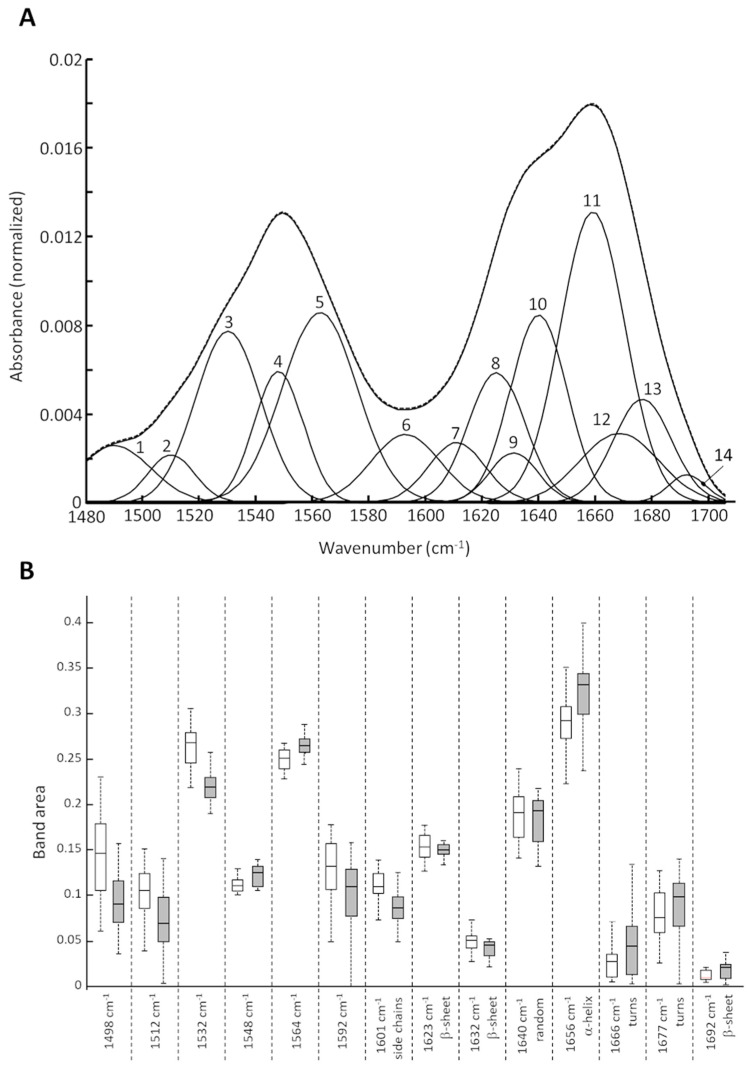
(**A**) Plot of the calculated subbands together with the resulting envelope and the original centroid spectrum. (**B**) Box-whisker plot of the calculated band area variations for GA-fixed (white bars) and SULEEI-treated (grey bars) samples. GA—Glutaraldehyde, SULEEI—Stabilization and sterilization of acellular pericardial scaffolds combining photo-initiated Ultraviolet cross-linking with Low-Energy Electron Irradiation.

As amide bands, here represented by the centroid spectra, are a complex unresolved line shape of vibration modes of the secondary structures, a curve fit analysis was performed to extract detailed information about the protein structure. The curve fitting algorithm resolved overlapped bands into distinct subbands and estimated parameters such as the band position, width, and area. Specific protein secondary structures have different vibration modes and thus absorb at specific wavelengths [34]. The curve fitting approach resulted in 14 subbands (Figure 3A). The estimated parameters and their correlation to protein secondary structures are summarized in Table S1.

The 14 subbands were assigned to secondary structure elements in the amide I region. Figure 3B shows the distribution of the band areas of all five bovine pericardia for GA-fixed (white boxes) and SULEEI-treated samples (grey boxes). The parameters of each subband are summarized in Table S1. Especially the band areas of the subbands located at 1532 cm^−1^ (amino groups), 1601 cm^−1^ (side chains), and 1656 cm^−1^ (α-helix) showed remarkable differences between the GA-fixed and SULEEI-treated samples (Figure 3B).

**Figure 4:**
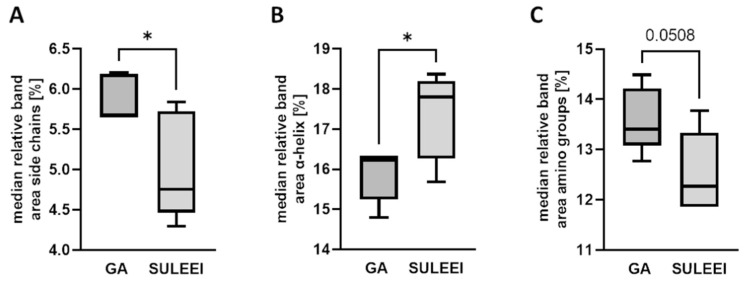
Box–whisker plots representing the differences in relative band area between GA-fixed and SULEEI-treated samples for side chains (**A**) and α-helix (**B**) and amino groups (**C**). *n* = 5. Unpaired *t*-test. * *p* ≤ 0.05. GA—Glutaraldehyde, SULEEI—Stabilization and sterilization of acellular pericardial scaffolds combining photo-initiated Ultraviolet cross-linking with Low-Energy Electron Irradiation.

The relative band area (*A_rel_*) of each subband was calculated using Equation (1). According to the pixel distribution for each subband, the median of *A_rel_* was determined for each individual GA-fixed and SULEEI-treated sample. The medians of the relative band areas were then compared between GA-fixed and SULEEI-treated samples (Figure 4 and Figure S1).

(1) $$A_{rel}\left(j\right)=\frac{A_{subband}\left(j\right)}{\sum_{j=1}^{subbands}A\left(j\right)}*100$$

Significant differences or at least a trend between GA-fixed and SULEEI-treated samples were detected for the subbands at 1532 cm^−1^ (amino groups, GA: 13.60 ± 0.65% vs. SULEEI: 12.53 ± 0.81%, *p* = 0.0508, *n* = 5), 1601 cm^−1^ (side chains, GA: 5.87 ± 0.29% vs. SULEEI: 5.03 ± 0.66%, *p* = 0.0322, *n* = 5), and 1656 cm^−1^ (α-helix, GA: 15.88 ± 0.66% vs. SULEEI: 17.35 ± 1.09%, *p* = 0.0325, *n* = 5). The results of the significance test are represented in Figure 4. While GA-treated samples had significant higher band areas for side chains (Figure 4A), SULEEI-treated samples were characterized by higher α-helical substructures (Figure 4B). Band areas for amino groups had a tendency to higher values in GA-fixed samples (Figure 4C). Consequently, the following analysis focused on these three subbands, as they showed remarkable differences between the two sample sets. Subbands that were not considered for further analysis are summarized in Figure S1. Although significant differences were also detected for subbands at 1548 cm^−1^ (*p* = 0.0114, *n* = 5) and 1692 cm^−1^ (antiparallel β-sheet; *p* = 0.0281, *n* = 5) (Figure S1), due to the low band area of subband 1692 cm^−1^ and the worse reflection of curve trend towards band wavelength 1548 cm^−1^, these two subbands were not considered for further analysis.

**Figure 5:**
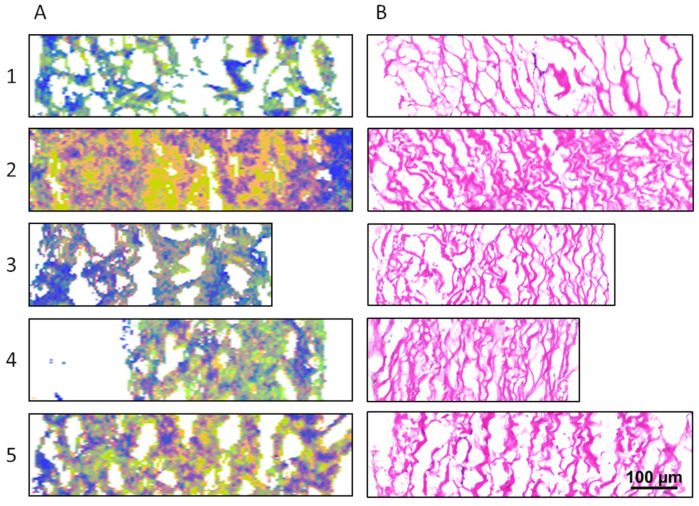
(**A**) RGB images of GA-fixed samples. The red channel reflects band areas of 1532 cm^−1^ (amino groups), the green channel visualizes band areas of 1601 cm^−1^ (side chains), and the blue channel represents band areas of 1656 cm^−1^ (α-helix). (**B**) H&E sections parallel stained to RGB images. Numbers 1–5 represent biological replicates of the bovine pericardium. RGB—Red—Green—Blue, GA—Glutaraldehyde, H&E—Hematoxylin and Eosin.

**Figure 6:**
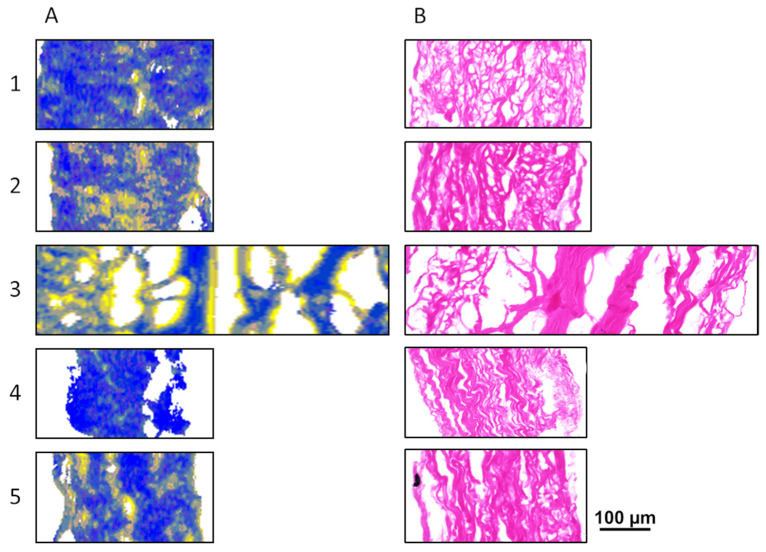
(**A**) RGB images of SULEEI-treated samples. The red channel reflects band areas of 1532 cm^−1^ (amino group), the green channel visualizes band areas of 1601 cm^−1^ (side chains), and the blue channel represents band areas of 1656 cm^−1^ (α-helix). (**B**) H&E sections parallel stained to RGB images. Numbers 1–5 represent biological replicates of the bovine pericardium. RGB—Red—Green—Blue, SULEEI—Stabilization and sterilization of acellular pericardial scaffolds combining photo-initiated Ultraviolet cross-linking with Low-Energy Electron Irradiation, H&E—Hematoxylin and Eosin.

These three substructure units with the most remarkable differences between GA-fixed and SULEEI-treated samples were then combined and transferred into RGB images. The red channel reflects band areas of 1532 cm^−1^ (amino groups), the green channel visualizes band areas of 1601 cm^−1^ (side chains), and the blue channel represents band areas of 1656 cm^−1^ (α-helix). Detailed information about differences between GA-fixed and SULEEI-treated samples, but also about the homogeneity or heterogeneity within the samples, can be derived from the RGB images. Consecutive sections were stained by H&E in order to correlate the RGB images to the sample morphology (Figure 5B and Figure 6B).

Figure 5 shows RGB and H&E-stained images of the GA-fixed samples, which reveal a wavy collagen structure with aligned collagen fibers (Figure 5B). The collagen fibers were less tightly packed in comparison with the SULEEI-treated samples, resulting in larger pericardium cross-sections for GA-fixed samples (Figure 6B). The RGB images were dominated by green and orange pixels, indicating high contents of side chains and amino groups. Blue pixels were only marginal present at distinct regions, indicating the appearance of α-helical substructures with a lower frequency compared with amino groups and side chains (Figure 5A).

The RGB images of SULEEI-treated samples are predominated by blue pixels, indicating a high content of α-helical substructures (Figure 6A). Additionally, yellow pixels are present at distinct regions, especially at the boundary regions of SULEEI-sample 3. Similar to GA-fixed samples, yellow pixels indicate almost balanced and high contents of amino groups and side chains. 

While orange pixels in GA-fixed samples represent a high content of amino groups (Figure 5A), the yellow pixels in SULEEI-treated samples indicate a high content of side chains (Figure 6A).

In comparison, GA-fixed tissue samples showed a more heterogeneous distribution of the three substructure elements throughout the tissue sections (Figure 5A) whereas SULEEI-treated tissue samples were more homogeneous, with predominantly blue pixels, indicating a high content of α-helical substructures (Figure 6A). Furthermore, SULEEI-treated pericardia exhibited a significantly reduced thickness of 0.284 ± 0.034 mm compared with GA-fixed counterparts (0.629 ± 0.153 mm) due to the drying process in filter paper before electron irradiation [25]. As a consequence, SULEEI-treated tissue samples (Figure 6B) are more compressed and show a less wavy and more densely aligned collagen structure than the GA-fixed samples. Therefore, entire SULEEI cross-sections result in smaller scan areas in comparison with the GA-fixed tissue samples (Figure 5A). Only SULEEI-treated-sample 3 (Figure 6B) showed a less compressed matrix structure similar to the GA-fixed tissue.

In summary, SULEEI-treated samples exhibited a higher content of α-helical substructures but less side chains and amino groups than the GA-fixed tissue samples. Therefore, a negative correlation between amino groups and side chains with α-helical substructures was detected.

## 4. Discussion

The development and investigation of alternative preparation strategies for cardiac implants is an important research focus, as conventionally GA-fixed bioprostheses are prone to calcification and patients thus need re-operation after 10–15 years [6,7,8,9,10,11]. It is suggested that the protein secondary structure is implicated in calcification processes [30]. In this study, bovine pericardium processed by an alternative preparation strategy, the so-called SULEEI-procedure, was analyzed by IR spectroscopic imaging combined with histological routine staining and was compared to conventionally GA-fixed tissue, especially regarding the chemical substructures.

The SULEEI-treated tissue samples showed a homogenous distribution of α-helical substructures. In contrast, GA-fixed tissue samples were more heterogeneous with locally high signals of side chains and amino groups. For other substructures, such as turns, random structures, and parallel β-sheets, no significant differences between GA-fixed and SULEEI-treated tissue samples were detected. Antiparallel β-sheet structures were significantly lower in the GA-fixed tissue samples, but due to the very low band area of this subband, this was not considered as a reliable result.

The locally high occurrence of side chains and amino groups in GA-fixed tissue samples reflects a high content of polar groups, which can act as crystallization nuclei at which the mineralization process is triggered, e.g., for the crystallization of calciumhydroxyapatite. Indeed, initially, the in vitro experiments using simulated body fluids for 18 days revealed a significantly reduced calcium content for SULEEI-treated samples compared with native counterparts, and will be further under investigation. The repeated preparation of five individual bovine pericardia revealed comparable distributions of secondary substructures by testing the means of the median relative band areas for amino groups, side chains, and α-helical substructures. They differ tendentially for amino groups (*p* = 0.0508) and significantly for side chains (*p* = 0.0322) and α-helical substructures (*p* = 0.0325) from the GA-fixation protocol. The GA-fixation protocol adopted in the present study is part of a patented protocol used for industrial heart valve prostheses [35]. The initial incubation step of 3h in 0.625% GA in HEPES (with addition of MgCl_2_ and NaCl) described therein was executed in the present study. In the patented protocol, the pericardium was subsequently further incubated in lower-concentrated GA for several days or even weeks. Therefore, the short incubation time in this study could be the cause for an incomplete crosslinking. In the chemical reaction of GA crosslinking, the free aldehyde groups of GA reacted with amine groups of lysine or hydroxylysine residues of proteins and formed a Schiff base intermediate. Aldol condensation of the Schiff bases led to a polymerization and finally to a crosslinking [36]. If these crosslinking reactions took place incompletely, then free aldehyde groups of GA and free amine groups of collagen remained and could act as a crystallization seed for the calcification process.

In contrast, bovine pericardium treated by the innovative SULEEI-procedure is rich in the collagen-typical α-helical substructure, indicating that this crosslinking protocol preserves the protein secondary structure. The amine and side chain content was lower in comparison to the GA-fixed tissue samples, and thus a lower susceptibility to calcification and in turn a longer valve durability is suggested. Nevertheless, the reduced crosslinking rate may be causal. As published before for porcine pericardium [12], in bovine pericardium, degradability in SULEEI-treated samples is increased in an in vitro collagen digestion assay [13]. However, the mechanical stability in the uniaxial tensile tests was equivalent [13,25]. Therefore, the impact of crosslinking efficacy on the IR spectra was not assumed. The SULEEI-protocol with its preservation of the α-helical substructure of collagen represented a promising alternative to GA-fixation for cardiac implants. Further evaluation of the SULEEI-treated material, especially concerning in vivo compatibility and mechanical behavior under physiological conditions, is envisioned.

Part of the SULEEI protocol is a decellularization step implementing not only RNAse and DNAse, but also trypsin [12,13]. Besides the efficient elimination of DNA below levels under 15 ng dsDNA/mg tissue dry weight and a complete absence of nuclei in histological evaluation [37] as postulated in Crapo et al. [15], this decellularization process can also be responsible for a reduction in GAG content. As the preservation of the matrix structure with its components is important for material stability and mechanical function, the further adaption of the decellularization step is an objective in ongoing experimental setups using more gentle protocol settings. Besides biomechanics and degradability, IR spectroscopy data also give information to correlate loss of amine content to material applicability—also observed in the presented setup—but only after a decellularization strategy in the SULEEI-pericardium. Degradation by irradiation steps can be excluded for SULEEI-treated samples, as quantitative biochemical assays do not show a significant reduction of hydroxyproline and elastin content compared with native counterparts. IR spectroscopy is suggested for biochemical evaluation of multiple tissue pathologies, such as cancer and fibrotic heart tissue, especially collagen [38,39]. Wang et al. investigated decellularized heart valve scaffolds regarding protein structure and solvent accessibility using IR spectroscopy [40]. They found that leaflet material contains a relatively high contribution of α-helical structures whereas the artery matrix contains a higher content of triple-helix and β-sheet structures. This confirms that the higher content of the α-helical substructure in the SULEEI-treated tissue samples is favorable.

Richards et al. performed IR spectroscopy to spatially analyze mineral properties as a function of disease progression on human aortic valves with calcific aortic valve disease [41]. Their results showed that apatite crystallinity increased, whereas the carbonate content decreased with the increasing valve calcification. In addition to the spectroscopic analysis of the protein secondary structure in the amide I region as presented in this study, the assessment of the carbonate/phosphate ratio of tissue samples could give important information about the state of calcification. Therefore, future experiments will include the IR spectroscopic analysis of SULEEI-treated tissue incubated in a bioreactor under physiological conditions. Furthermore, to investigate the calcification potential after in vivo implantation, IR spectroscopy has to be performed on explanted SULEEI-tissue and analyzed especially for the phosphate/carbonate ratio. The results of the IR spectroscopic imaging will be correlated to the quantitative calcium assays, as well as histological staining for mineralization by von Kossa.

In this study, it was shown that the innovative SULEEI-preparation preserves the typical α-helical substructure of collagen in bovine pericardium, whereas GA-fixed tissue samples are higher in polar groups acting as crystallization points for mineralization. The chemical structure of SULEEI-treated bovine pericardium is thus superior to GA-fixed tissue, especially for application in cardiac surgery, as these results suggest a lower calcification potential.

## 5. Conclusions

In this study, the structural characterization of an alternative GA-free preparation method, named SULEEI, for the preparation of bovine pericardium for application in cardiac surgery was performed using IR spectroscopic imaging combined with multivariate data and curve fit analysis. In detail, the protein secondary structure was investigated and compared to conventionally GA-fixed bovine pericardium, as it is implicated in calcification processes. SULEEI-treated pericardial tissue was high in α-helix, which is the most prominent secondary structure of collagen. In contrast, the GA-fixed tissue exhibited high contents of amino groups and side chains, leading to an increased occurrence of polar groups acting as nucleation points for mineralization processes. Therefore, IR spectroscopic imaging could be successfully applied to estimate the calcification potential of innovative biomaterials. SULEEI-treated bovine pericardium is expected to be less prone to calcification in comparison with GA-fixed tissue and in turn could be a promising alternative for application in cardiac surgery.

## Figures and Tables

**Table 1:** SULEEI treatment of bovine pericardium. In the first step, the pericardia were decellularized (DECELL), in the second step they were pre-treated with riboflavin/dextran solution and crosslinked by UV-light for stabilization (STABI), and in the third step they were sterilized by low-energy electron irradiation (STERI).

DECELL	1% Triton X-100 (Carl Roth GmbH & Co. KG, Karlsruhe, Germany) in 5 mM TRIS (pH 8), 4 °C, 8 h
	2× Hank’s BSS with Ca^2+^ and Mg^2+^ (Carl Roth GmbH & Co. KG, Karlsruhe, Germany), 15 min, 4 °C
	0.1 mg/mL DNAse, 0.02 mg/mL RNAse (both from Serva Electrophoresis GmbH, Heidelberg, Germany), 0.004 mg/mL trypsine (Biochrom GmbH, Berlin, Germany) in HBSS, 37 °C, 15 h
	1% Triton X-100 in 5 mM TRIS (pH 8), 4 °C, 7 h
	2–4 × PBS, 30 min
STABI	2% dextran T500 (Carl Roth GmbH & Co. KG, Karlsruhe, Germany) in PBS, 0.1% riboflavin (Serva Electrophoresis GmbH, Heidelberg, Germany), 4 °C, 1 h
	UVA-irradiation at 320–480 nm (Bio-Link 254, Vilber Lourmat GmbH, Eberhardzell, Germany) with 0.3 mW/cm^2^, 3 h, fibrous and serous side
	4 × PBS, 30 min
STERI	Dehydration process to semi-dry status by incubation in filter paper, 10 min
	Transfer into HDPE (high density polyethylen) foil
	Low-energy electron irradiation, atmospheric pressure, accelerating voltage of 200 kV, current of 3 mA, dose of 36 kGy, fibrous, and serous side, (KeVac System, Linac Technologies, Orsay, France (200 kV, 5 mA))

## Data Availability

Data available upon request. Please contact the coresponding author.

## Supplementary Materials

The following supporting information can be downloaded at: https://www.mdpi.com/article/10.3390/life12122035/s1, Table S1: Properties of subbands after curve fitting. Subbands that were analyzed in more detail are marked in grey. Figure S1: Box–whisker plots representing statistically non significant differences in relative band area between GA-fixed and SULEEI-treated samples. These subbands were not analyzed further. *n* = 5. Unpaired *t*-test. * *p* ≤ 0.05, ns = not significant.
