# Supplementary material for: Infrared Spectroscopic Verification of a α-Helical Collagen Structure in Glutaraldehyde-Free Crosslinked Bovine Pericardium for Cardiac Implants
Source: Life (Basel). 2022 Dec 6;12(12):2035. doi: 10.3390/life12122035 (PMC9785276; doi:10.3390/life12122035)
Supplement: Supplementary file 1 [file life-12-02035-s001.zip › life-2041056-supplementary.pdf]

Supporting Information

**Table S1.** Properties of subbands after curve fitting. Subbands that were analyzed in more detail are marked in grey

| Sub band | Assign ment | Mean center ± STD | | Mean area ± STD | | Mean width ± STD | | Percentage | |
|---|---|---|---|---|---|---|---|---|---|
| | | GA | SULEEI | GA | SULEEI | GA | SULEEI | GA | SULEEI |
| 1 | | 1490.1 ± 0.1 | 1490.0 ± 0.0 | 0.145 ± 0.062 | 0.114 ± 0.062 | 29.985 ± 0.025 | 29.986 ± 0.015 | 7.7 % | 5.7 % |
| 2 | | 1510.7 ± 0.4 | 1510.7 ± 0.4 | 0.098 ± 0.031 | 0.076 ± 0.035 | 21.273 ± 0.946 | 20.531 ± 1.208 | 5.3 % | 3.9 % |
| 3 | amino groups | 1530.2 ± 0.2 | 1530.2 ± 0.3 | 0.259 ± 0.027 | 0.238 ± 0.034 | 26.831 ± 0.679 | 26.968 ± 0.817 | 13.7 % | 12.3 % |
| 4 | | 1548.9 ± 0.7 | 1548.5 ± 0.7 | 0.113 ± 0.009 | 0.130 ± 0.022 | 21.042 ± 0.783 | 21.488 ± 0.840 | 5.8 % | 6.7 % |
| 5 | | 1565.4 ± 2.5 | 1564.3 ± 2.3 | 0.250 ± 0.013 | 0.272 ± 0.021 | 34.850 ± 2.007 | 32.591 ± 1.994 | 13.1 % | 14.1 % |
| 6 | | 1595.0 ± 0.9 | 1593.8 ± 1.4 | 0.125 ± 0.035 | 0.116 ± 0.028 | 27.791 ± 2.183 | 28.521 ± 1.715 | 6.7 % | 6.0 % |
| 7 | side chains | 1610.7 ± 0.8 | 1610.5 ± 0.4 | 0.109 ± 0.019 | 0.093 ± 0.026 | 26.522 ± 1.646 | 25.646 ± 2.448 | 5.7 % | 4.8 % |
| 8 | β-sheet | 1622.2 ± 1.2 | 1623.2 ± 1.3 | 0.164 ± 0.018 | 0.154 ± 0.008 | 24.741 ± 1.323 | 24.348 ± 0.901 | 8.4 % | 8.1 % |
| 9 | β-sheet | 1629.6 ± 1.7 | 1629.9 ± 1.4 | 0.045 ± 0.018 | 0.043 ± 0.013 | 19.442 ± 2.751 | 19.430 ± 1.833 | 2.3 % | 2.3 % |
| 10 | random | 1638.7 ± 0.7 | 1639.1 ± 0.5 | 0.190 ± 0.031 | 0.195 ± 0.026 | 24.082 ± 1.228 | 23.973 ± 1.154 | 9.7 % | 10.2 % |
| 11 | α-helix | 1657.1 ± 0.7 | 1657.7 ± 0.3 | 0.300 ± 0.057 | 0.324 ± 0.06 | 27.677 ± 1.318 | 27.422 ± 0.893 | 15.6 % | 17.1 % |
| 12 | turns | 1667.4 ± 0.6 | 1667.8 ± 0.4 | 0.031 ± 0.029 | 0.053 ± 0.044 | 17.456 ± 8.031 | 21.401 ± 8.974 | 1.3 % | 2.8 % |
| 13 | turns | 1676.0 ± 2.2 | 1677.1 ± 1.4 | 0.079 ± 0.029 | 0.053 ± 0.044 | 23.029 ± 3.404 | 22.747 ± 2.433 | 4.2 % | 4.9 % |
| 14 | β-sheet | 1692.3 ± 1.6 | 1692.2 ± 1.4 | 0.012 ± 0.006 | 0.097 ± 0.027 | 21.793 ± 6.911 | 16.649 ± 3.857 | 0.6 % | 1.0 % |

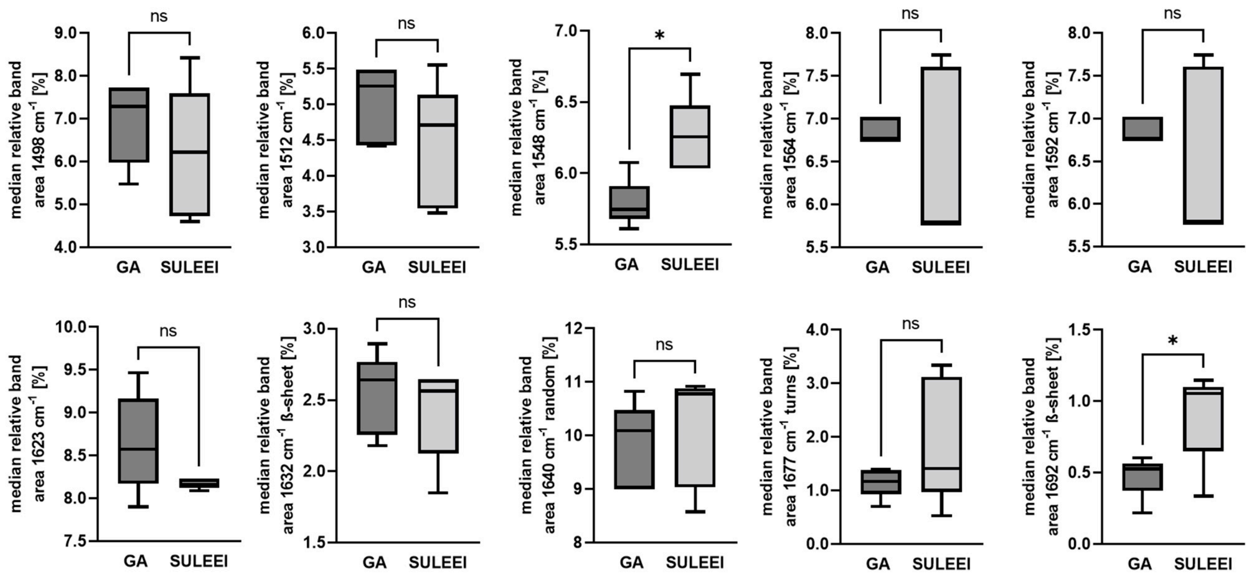


**Figure S1.** Box-whisker plots representing statistical not significant differences in relative band area between GA-fixed and SULEEI-treated samples. These subbands were not analyzed further. n=5. Unpaired t-test. *p≤0.05, ns = not significant.
